# CONSORT 2010 statement: Updated guidelines for reporting parallel group randomised trials

**DOI:** 10.4103/0976-500X.72352

**Published:** 2010

**Authors:** Kenneth F. Schulz, Douglas G. Altman, David Moher

**Affiliations:** *Family Health International, Research Triangle Park, North Carolina, United States of America*; 1 *Centre for Statistics in Medicine, University of Oxford, Wolfson College, Oxford, United Kingdom*; 2 *Ottawa Methods Centre, Clinical Epidemiology Program, Ottawa Hospital Research Institute, Department of Epidemiology and Community Medicine, University of Ottawa, Ottawa, Canada*

## INTRODUCTION

Randomised controlled trials, when appropriately designed, conducted and reported, represent the gold standard in evaluating healthcare interventions. However, randomised trials can yield biased results if they lack methodological rigour.[1] To assess a trial accurately, readers of a published report need complete, clear and transparent information on its methodology and findings. Unfortunately, attempted assessments frequently fail because authors of many trial reports neglect to provide lucid and complete descriptions of that critical information.[2–4]

That lack of adequate reporting fuelled the development of the original CONSORT (Consolidated Standards of Reporting Trials) statement in 1996[5] and its revision five years later.[6–8] While those statements improved the reporting quality for some randomised controlled trials,[9,10] many trial reports still remain inadequate.[2] Furthermore, new methodological evidence and additional experience has accumulated since the last revision in 2001. Consequently, we organised a CONSORT Group meeting to update the 2001 statement.[6–8] We introduce here the result of that process, CONSORT 2010.

## INTENT OF CONSORT 2010

**Figure 1:**
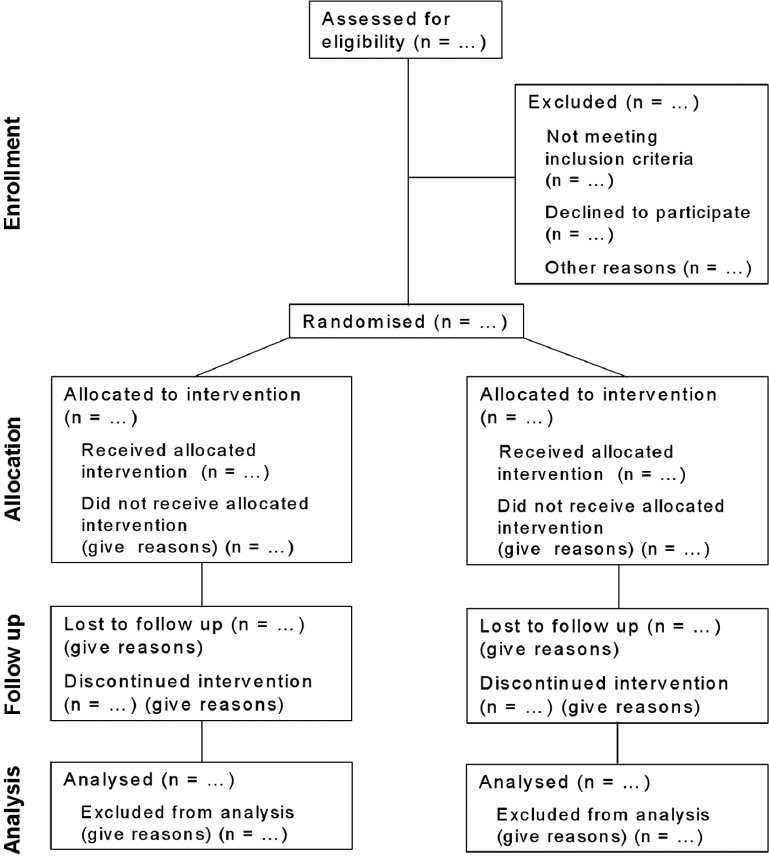
Flow diagram of the progress through the phases of a parallel randomised trial of two groups (that is, enrolment, intervention allocation, follow-up and data analysis).

The CONSORT 2010 Statement is this paper including the 25 item checklist in the [Table 1] and the flow diagram [Figure 1]. It provides guidance for reporting all randomised controlled trials, but focuses on the most common design type—individually randomised, two group, parallel trials. Other trial designs, such as cluster randomised trials and non-inferiority trials, require varying amounts of additional information. CONSORT extensions for these designs,[11,12] and other CONSORT products, can be found through the CONSORT website (http://www.consort-statement.org). Along with the CONSORT statement, we have updated the explanation and elaboration article,[13] which explains the inclusion of each checklist item, provides methodological background and gives published examples of transparent reporting.

**Table 1:** CONSORT 2010 checklist of information to include when reporting a randomised trial (for a downloadable version of this checklist see Text S1 or the CONSORT website)*

Section/Topic	Item no.	Checklist Item	Reported on page no.
Title and abstract			
	1a	Identification as a randomised trial in the title	
	1b	Structured summary of trial design, methods, results and conclusions (for specific guidance see CONSORT for abstracts[21,31])	
Introduction			
Background and objectives	2a	Scientific background and explanation of rationale	
	2b	Specific objectives or hypotheses	
Methods			
Trial design	3a	Description of trial design(such as parallel, factorial) including allocation ratio	
	3b	Important charges to methods after trial commencement (such as eligibility criteria), with reasons	
Participations	4a	Eligibility criteria for participants	
	4b	Settings and locations where the data were collected	
Interventions	5	The interventions for each group with sufficient details to allow replication, including how and when they were actually administered	
Outcomes	6a	Completely defined pre-specified primary and secondary outcome measures, including how and when they were assessed	
	6b	Any changes to trial outcomes after the trial commenced, with reasons	
Sample size	7a	How sample size was determined	
	7b	When applicable, explanation of any interim analyses and stopping guidelines	
Randomisation			
Sequence generation	8a	Method used to generate the random allocation sequence	
	8b	Type of randomisation, details of any restriction (such as blocking and block size)	
Allocation concealment mechanism	9	Mechanism used to implement the random allocation sequence (such as sequentially numbered containers), describing any steps taken to conceal the sequence until interventions were assigned	
Implementation	10	Who generated the random allocation sequence, who enrolled participants, and who assigned participants to interventions	
Blinding	11a	If done, who was blinded after assignment to interventions (for example, participants, care providers, those assessing outcomes) and how	
	11b	If relevant, description of the similarity of interventions	
Statistical methods	12a	Statistical methods used to compare groups for primary and secondary outcomes	
	12b	Methods for additional analyses, such as subgroup analyses and adjusted analyses
Results			
Participant flow (a diagram is strongly recommended)	13a	For each group, the numbers of participants who were randomly assigned, received intended treatment, and were analysed for primary outcome	
	13b	For each group, losses and exclusions after randomisation, together with reasons
Recruitment	14a	Dates defining the periods of recruitment and follow-up	
	14b	Why the trial ended or was stopped	
Baseline data	15	A table showing baseline demographic and clinical characteristics for each group	
Number analysed	16	For each group, number of participants (denominator) included in each analysis and whether the analysis was by original assigned groups
Outcomes and estimation	17a	For each primary and secondary outcomes, results for each group, and the estimated effect size and its precision (such as 95% confidence interval)	
	17b	For binary outcomes, presentation of both absolute and relative effect sizes in recommended
Ancillary analyses	18	Results of any other analyses perform, including subgroup analyses and adjusted analyses, distinguishing pre-specified from exploratory	
Harms	19	All important harms or unintended effects in each group (for specific guidance see CONSORT for harms[28])
Discussion			
Limitations	20	Trial limitations, addressing sources of potential bias, imprecision, and, if relevant, multiplicity of analyses	
Generalisability	21	Generalisability (external validity, applicability) of trial findings	
Interpretation	22	Interpretation consistent with results, balancing benefits and harms, and considering other relevant evidence
Other information			
Registration	23	Registration number and name of trial registry
Protocol	24	Where the full trial protocol can be accessed, if available
Funding	25	Sources of funding and other support (such as supply of drugs), role of funders

* We strongly recommend reading this statement in conjunction with the CONSORT 2010 Explanation and Elaboration[13] for important clarifications, on all the items. If relevant, we also recommend reading CONSORT extensions for cluster randomised trials[11], non-inferiority and equivalence trials[12], nonpharmacological treatments[32], herbal interventions[33], and pragmatic trials[34]. Additional extensions are forthcoming: for those and for up to date references relevant to this checklist, see http://www.consort-statement.org

Diligent adherence by authors to the checklist items facilitates clarity, completeness and transparency of reporting. Explicit descriptions, not ambiguity or omission, best serve the interests of all readers. Note that the CONSORT 2010 Statement does not include recommendations for designing, conducting and analysing trials. It solely addresses the reporting of what was done and what was found.

Nevertheless, CONSORT does indirectly affect design and conduct. Transparent reporting reveals deficiencies in research if they exist. Thus, investigators who conduct inadequate trials, but who must transparently report, should not be able to pass through the publication process without revelation of their trial’s inadequacies. That emerging reality should provide impetus to improved trial design and conduct in the future, a secondary indirect goal of our work. Moreover, CONSORT can help researchers in designing their trial.

## BACKGROUND TO CONSORT

Efforts to improve the reporting of randomised controlled trials accelerated in the mid-1990s, spurred partly by methodological research. Researchers had shown for many years that authors reported such trials poorly and empirical evidence began to accumulate that some poorly conducted or poorly reported aspects of trials were associated with bias[14] Two initiatives aimed at developing reporting guidelines culminated in one of us (DM) and Drummond Rennie organising the first CONSORT statement in 1996.[5] Further methodological research on similar topics reinforced earlier findings[15] and fed into the revision of 2001.[6–8] Subsequently, the expanding body of methodological research informed the refinement of CONSORT 2010. More than 700 studies comprise the CONSORT database (located on the CONSORT website), which provides the empirical evidence to underpin the CONSORT initiative.

Indeed, CONSORT Group members continually monitor the literature. Information gleaned from these efforts provides an evidence base on which to update the CONSORT statement. We add, drop, or modify items based on that evidence and the recommendations of the CONSORT Group, an international and eclectic group of clinical trialists, statisticians, epidemiologists and biomedical editors. The CONSORT Executive (KFS, DGA, DM) strives for a balance of established and emerging researchers. The membership of the group is dynamic. As our work expands in response to emerging projects and needed expertise, we invite new members to contribute. As such, CONSORT continually assimilates new ideas and perspectives. That process informs the continually evolving CONSORT statement.

Over time, CONSORT has garnered much support. More than 400 journals, published around the world and in many languages, have explicitly supported the CONSORT statement. Many other healthcare journals support it without our knowledge. Moreover, thousands more have implicitly supported it with the endorsement of the CONSORT statement by the International Committee of Medical Journal Editors (http://www.icmje.org). Other prominent editorial groups, the Council of Science Editors and the World Association of Medical Editors, officially support CONSORT. That support seems warranted: when used by authors and journals, CONSORT seems to improve reporting.[9]

## DEVELOPMENT OF CONSORT 2010

Thirty one members of the CONSORT 2010 Group met in Montebello, Canada, in January 2007 to update the 2001 CONSORT statement. In addition to the accumulating evidence relating to existing checklist items, several new issues had come to prominence since 2001. Some participants were given primary responsibility for aggregating and synthesising the relevant evidence on a particular checklist item of interest. Based on that evidence, the group deliberated the value of each item. As in prior CONSORT versions, we kept only those items deemed absolutely fundamental to reporting a randomised controlled trial. Moreover, an item may be fundamental to a trial but not included, such as approval by an institutional ethical review board, because funding bodies strictly enforce ethical review and medical journals usually address reporting ethical review in their instructions for authors. Other items may seem desirable, such as reporting on whether on-site monitoring was done, but a lack of empirical evidence or any consensus on their value cautions against inclusion at this point. The CONSORT 2010 Statement thus addresses the minimum criteria, although that should not deter authors from including other information if they consider it important.

After the meeting, the CONSORT Executive convened teleconferences and meetings to revise the checklist. After seven major iterations, a revised checklist was distributed to the larger group for feedback. With that feedback, the executive met twice in person to consider all the comments and to produce a penultimate version. That served as the basis for writing the first draft of this paper, which was then distributed to the group for feedback. After consideration of their comments, the executive finalised the statement.

The CONSORT Executive then drafted an updated explanation and elaboration manuscript, with assistance from other members of the larger group. The substance of the 2007 CONSORT meeting provided the material for the update. The updated explanation and elaboration manuscript was distributed to the entire group for additions, deletions and changes. That final iterative process converged to the CONSORT 2010 Explanation and Elaboration.[13]

## CHANGES IN CONSORT 2010

The revision process resulted in evolutionary, not revolutionary, changes to the checklist [Table 1] and the flow diagram was not modified except for one word [Figure 1]. Moreover, because other reporting guidelines augmenting the checklist refer to item numbers, we kept the existing items under their previous item numbers except for some renumbering of items 2 to 5. We added additional items either as a sub-item under an existing item, an entirely new item number at the end of the checklist, or (with item 3) an interjected item into a renumbered segment. We have summarised the noteworthy general changes in Box 1 and specific changes in Box 2. The CONSORT website contains a side by side comparison of the 2001 and 2010 versions.

**Box 1.**

Noteworthy General Changes in CONSORT 2010 StatemeWe simplified and clarified the wording, such as in items 1, 8, 10, 13, 15, 16, 18, 19 and 21.

We improved consistency of style across the items by removing the imperative verbs that were in the 2001 version.

We enhanced specificity of appraisal by breaking some items into sub-items. Many journals expect authors to complete a CONSORT checklist indicating where in the manuscript the items have been addressed. Experience with the checklist noted pragmatic difficulties when an item comprised multiple elements. For example, item 4 addresses eligibility of participants and the settings and locations of data collection. With the 2001 version, an author could provide a page number for that item on the checklist, but might have reported only eligibility in the paper, for example and not reported the settings and locations. CONSORT 2010 relieves obfuscations and forces authors to provide page numbers in the checklist for both eligibility and settings.

**Box 2.**

Noteworthy Specific Changes in CONSORT 2010 Statement*Item 1b (title and abstract)* – We added a sub-item on providing a structured summary of trial design, methods, results and conclusions and referenced the CONSORT for abstracts article.[21]

*Item 2b (introduction)* – We added a new sub-item (formerly item 5 in CONSORT 2001) on “Specific objectives or hypotheses”.

*Item 3a (trial design)* – We added a new item including this sub-item to clarify the basic trial design (such as parallel group, crossover, cluster) and the allocation ratio.

*Item 3b (trial design)* – We added a new sub-item that addresses any important changes to methods after trial commencement, with a discussion of reasons.

*Item 4 (participants)* – Formerly item 3 in CONSORT 2001.

*Item 5 (interventions)* – Formerly item 4 in CONSORT 2001. We encouraged greater specificity by stating that descriptions of interventions should include “sufficient details to allow replication”.[3]

*Item 6 (outcomes)* – We added a sub-item on identifying any changes to the primary and secondary outcome (endpoint) measures after the trial started. This followed from empirical evidence that authors frequently provide analyses of outcomes in their published papers that were not the prespecified primary and secondary outcomes in their protocols, while ignoring their prespecified outcomes (that is, selective outcome reporting).[4,22] We eliminated text on any methods used to enhance the quality of measurements.

*Item 9 (allocation concealment mechanism)* – We reworded this to include mechanism in both the report topic and the descriptor to reinforce that authors should report the actual steps taken to ensure allocation concealment rather than simply report imprecise, perhaps banal, assurances of concealment.

*Item 11 (blinding)* – We added the specification of how blinding was done and, if relevant, a description of the similarity of interventions and procedures. We also eliminated text on “how the success of blinding (masking) was assessed” because of a lack of empirical evidence supporting the practice as well as theoretical concerns about the validity of any such assessment.[23,24]

*Item 12a (statistical methods)* – We added that statistical methods should also be provided for analysis of secondary outcomes.

*Sub-item 14b (recruitment)* – Based on empirical research, we added a sub-item on “Why the trial ended or was stopped”.[25]

*Item 15 (baseline data)* – We specified “A table” to clarify that baseline and clinical characteristics of each group are most clearly expressed in a Table.

*Item 16 (numbers analysed)* – We replaced mention of “intention to treat” analysis, a widely misused term, by a more explicit request for information about retaining participants in their original assigned groups.[26]

*Sub-item 17b (outcomes and estimation)* – For appropriate clinical interpretability, prevailing experience suggested the addition of “For binary outcomes, presentation of both relative and absolute effect sizes is recommended”.[27]

*Item 19 (harms)* – We included a reference to the CONSORT paper on harms.[28]

*Item 20 (limitations)* – We changed the topic from “Interpretation” and supplanted the prior text with a sentence focusing on the reporting of sources of potential bias and imprecision.

*Item 22 (interpretation)* – We changed the topic from “Overall evidence.” Indeed, we understand that authors should be allowed leeway for interpretation under this nebulous heading. However, the CONSORT Group expressed concerns that conclusions in papers frequently misrepresented the actual analytical results and that harms were ignored or marginalised. Therefore, we changed the checklist item to include the concepts of results matching interpretations and of benefits being balanced with harms.

*Item 23 (registration)* – We added a new item on trial registration. Empirical evidence supports the need for trial registration and recent requirements by journal editors have fostered compliance.[29]

*Item 24 (protocol)* – We added a new item on availability of the trial protocol. Empirical evidence suggests that authors often ignore, in the conduct and reporting of their trial, what they stated in the protocol.[4,22] Hence, availability of the protocol can instigate adherence to the protocol before publication and facilitate assessment of adherence after publication.

*Item 25 (funding)* – We added a new item on funding. Empirical evidence points toward funding source sometimes being associated with estimated treatment effects.[30]

## IMPLICATIONS AND LIMITATIONS

We developed CONSORT 2010 to assist authors in writing reports of randomised controlled trials, editors and peer reviewers in reviewing manuscripts for publication and readers in critically appraising published articles. The CONSORT 2010 Explanation and Elaboration provides elucidation and context to the checklist items. We strongly recommend using the explanation and elaboration in conjunction with the checklist to foster complete, clear and transparent reporting and aid appraisal of published trial reports.

CONSORT 2010 focuses predominantly on the two group, parallel randomised controlled trial, which accounts for over half of trials in the literature.[2] Most of the items from the CONSORT 2010 Statement, however, pertain to all types of randomised trials. Nevertheless, some types of trials or trial situations dictate the need for additional information in the trial report. When in doubt, authors, editors and readers should consult the CONSORT website for any CONSORT extensions, expansions (amplifications), implementations, or other guidance that may be relevant.

The evidence based approach we have used for CONSORT also served as a model for development of other reporting guidelines, such as for reporting systematic reviews and meta-analyses of studies evaluating interventions,[16] diagnostic studies,[17] and observational studies.[18] The explicit goal of all these initiatives is to improve reporting. The Enhancing the Quality and Transparency of Health Research (EQUATOR) Network will facilitate development of reporting guidelines and help disseminate the guidelines: http://www.equator-network.org provides information on all reporting guidelines in health research.

With CONSORT 2010, we again intentionally declined to produce a rigid structure for the reporting of randomised trials. Indeed, SORT[19] tried a rigid format and it failed in a pilot run with an editor and authors.[20] Consequently, the format of articles should abide by journal style, editorial directions, the traditions of the research field addressed and, where possible, author preferences. We do not wish to standardise the structure of reporting. Authors should simply address checklist items somewhere in the article, with ample detail and lucidity. That stated, we think that manuscripts benefit from frequent subheadings within the major sections, especially the methods and results sections.

CONSORT urges completeness, clarity and transparency of reporting, which simply reflects the actual trial design and conduct. However, as a potential drawback, a reporting guideline might encourage some authors to report fictitiously the information suggested by the guidance rather than what was actually done. Authors, peer reviewers and editors should vigilantly guard against that potential drawback and refer, for example, to trial protocols, to information on trial registers and to regulatory agency websites. Moreover, the CONSORT 2010 Statement does not include recommendations for designing and conducting randomised trials. The items should elicit clear pronouncements of how and what the authors did, but do not contain any judgments on how and what the authors should have done. Thus, CONSORT 2010 is not intended as an instrument to evaluate the quality of a trial. Nor is it appropriate to use the checklist to construct a “quality score.”

Nevertheless, we suggest that researchers begin trials with their end publication in mind. Poor reporting allows authors, intentionally or inadvertently, to escape scrutiny of any weak aspects of their trials. However, with wide adoption of CONSORT by journals and editorial groups, most authors should have to report transparently all important aspects of their trial. The ensuing scrutiny rewards well conducted trials and penalises poorly conducted trials. Thus, investigators should understand the CONSORT 2010 reporting guidelines before starting a trial as a further incentive to design and conduct their trials according to rigorous standards.

CONSORT 2010 supplants the prior version published in 2001. Any support for the earlier version accumulated from journals or editorial groups will automatically extend to this newer version, unless specifically requested otherwise. Journals that do not currently support CONSORT may do so by registering on the CONSORT website. If a journal supports or endorses CONSORT 2010, it should cite one of the original versions of CONSORT 2010, the CONSORT 2010 Explanation and Elaboration and the CONSORT website in their “Instructions to authors.” We suggest that authors who wish to cite CONSORT should cite this or another of the original journal versions of CONSORT 2010 Statement and, if appropriate, the CONSORT 2010 Explanation and Elaboration.[13] All CONSORT material can be accessed through the original publishing journals or the CONSORT website. Groups or individuals who desire to translate the CONSORT 2010 Statement into other languages should first consult the CONSORT policy statement on the website.

We emphasise that CONSORT 2010 represents an evolving guideline. It requires perpetual reappraisal and, if necessary, modifications. In the future we will further revise the CONSORT material considering comments, criticisms, experiences and accumulating new evidence. We invite readers to submit recommendations via the CONSORT website.
